# Cognitive function and depressive symptoms in major depressive disorder: a smartphone-based study of outpatients from a sleep-disorders clinic in China

**DOI:** 10.3389/fpsyt.2026.1713792

**Published:** 2026-04-16

**Authors:** Ci Yan, Pan Yan, Zhenghe Yu, Junhang Zhang, Mingfen Song, Hongjing Mao

**Affiliations:** Affiliated Mental Health Center and Hangzhou Seventh People’s Hospital, Zhejiang University School of Medicine, Hangzhou, Zhejiang, China

**Keywords:** cognition, depressive disorder, major, mobile applications, sleep disorders, treatment outcome

## Abstract

**Background:**

Subjective cognitive dysfunction is common in patients with major depressive disorder (MDD), but its relationship with depressive symptoms and treatment response remains unclear, especially in routine clinical settings in China.

**Methods:**

We conducted an 8-week retrospective observational study using routinely collected data from the “Good Sleep 365” smartphone platform at the Sleep Disorders Diagnosis and Treatment Center of Hangzhou Seventh People’s Hospital (China) between 1 November 2017 and 10 October 2024. Adults aged 18–65 years who met ICD-10 criteria for a depressive episode, had baseline PHQ-9 ≥10, and completed the PDQ-D-20 were included. PHQ-9 and PDQ-D-20 were assessed at baseline and at weeks 2, 4, 6 and 8. Correlations between depressive symptoms and subjective cognition were examined. Among 233 patients who completed all follow-ups, three hierarchical logistic regression models were built to predict antidepressant response (PHQ-9 reduction ≥50% at week 8), and their performance was evaluated using ROC curves, calibration, decision curve analysis and a nomogram.

**Results:**

In 321 patients with MDD, higher PHQ-9 scores were consistently associated with more severe PDQ-D-20 total and domain scores at all time points. Higher baseline PDQ-D-20 scores predicted smaller reductions in PHQ-9. Baseline depressive and cognitive measures did not show marked associations with age, sex, education level, occupation or illness duration. The fully adjusted logistic model that combined baseline PDQ-D-20, symptom scales and demographic/clinical variables showed good discrimination (AUC ≈0.91) and acceptable calibration, and baseline PDQ-D-20 remained an independent predictor of non-response.

**Conclusion:**

In this smartphone-based cohort of Chinese outpatients from a sleep-disorders clinic, subjective cognitive dysfunction was closely related to depressive symptom burden and predicted poorer antidepressant response, suggesting that routine cognitive assessment may help identify patients at risk of suboptimal outcomes.

## 1 Introduction

Major depressive disorder (MDD) is one of the most common disabling diseases in the world today (1). The World Health Organization forecasts that depressive disorder will rank first in the disease burden by 2030 (2). Due to the high prevalence of MDD, 11% to 15% of the global population will be affected by MDD at some point in their lives (3), and MDD is prone to relapse, therefore, MDD should receive worldwide attention. In China, rapid social and economic change, high academic and occupational pressure, and persistent stigma surrounding mental illness may further increase the burden of depression, and many patients initially seek help for sleep or somatic complaints rather than mood symptoms.

The main clinical symptoms of MDD are persistent low mood, loss of pleasure, and decreased energy. In addition, there are also various degrees of cognitive impairment, including reduced cognitive flexibility, memory loss, attention loss, and impaired executive function (4). Cognitive impairment has a serious impact on MDD patients in study, work, and daily life. MDD patients have a complex relationship between cognitive function and emotional symptoms. Some studies suggest that the cognitive function of MDD is not related to the severity of depression symptoms and depression (5, 6). Some studies suggest that the cognitive function of MDD patients is positively correlated with the severity of depression, that is, the more severe the degree of depression, the more severe the cognitive impairment (7, 8). In clinical practice, we often encounter patients with depression complaining of their memory loss, attention loss, and inability to adapt well to their studies and work. To this day, the relationship between depression and cognitive function has not been conclusively determined. In the present study we focus on subjective cognitive dysfunction, defined as patients’ perceived difficulties in everyday cognitive activities. The 20-item Perceived Deficits Questionnaire for Depression, short form (PDQ-D-20), evaluates four key domains that are closely related to functioning in daily life, namely attention and concentration, retrospective memory, prospective memory, and planning and organization (9). Previous studies have shown that more severe cognitive symptoms, whether measured by objective neuropsychological tests or by self-report instruments such as the PDQ-D, are associated with greater depressive symptom burden, poorer psychosocial functioning and lower quality of life in patients with MDD (10, 11). Moreover, meta-analyses and longitudinal cohort studies indicate that cognitive impairment is not merely an epiphenomenon of low mood but represents a core dimension of MDD that may persist into remission and requires specific clinical attention (10, 12).

Therefore, further elucidating the relationship between the two will not only allow us to gain a deeper theoretical understanding of the etiology and pathological mechanism of MDD, but also provide clues and evidence for the comprehensive recovery of MDD in clinical diagnosis and treatment. In addition to cross-sectional associations, an emerging body of longitudinal research has suggested that higher baseline levels of subjective cognitive symptoms are linked to a lower probability of achieving symptomatic remission, functional recovery or sustained wellness during antidepressant treatment (11, 13). However, evidence from routine clinical practice in China, especially in patients presenting with prominent sleep complaints and followed repeatedly via smartphone-based platforms, remains limited, and the potential value of baseline subjective cognition for predicting treatment response has not been fully explored.

Based on this background, the present study used longitudinal data collected through the Good Sleep 365 smartphone platform in the sleep disorders diagnosis and treatment center of Hangzhou Seventh People’s Hospital to describe the relationship between depressive symptoms and subjective cognitive dysfunction over an eight-week period in outpatients with MDD. At the same time, we explored whether baseline subjective cognitive complaints are related to subsequent improvement in depressive symptoms under routine clinical treatment. This work is intended to supplement the lack of clinical evidence from Chinese outpatient settings, especially in patients with prominent sleep complaints followed via smartphone-based assessments, and to provide a preliminary basis for more refined assessment and intervention strategies in future research. We hypothesized that more severe baseline subjective cognitive dysfunction would be associated with higher depressive symptom severity at all time points and with a lower probability of achieving antidepressant treatment response over eight weeks. The present study is innovative in using a smartphone-based platform in a sleep-disorders clinic in China to repeatedly assess subjective cognition and depressive symptoms in routine practice and in applying multivariable logistic regression to quantify the added predictive value of cognitive complaints for short-term treatment outcome.

## 2 Materials and methods

### 2.1 Study design and participants

This retrospective observational study used routinely collected data from patients who attended the Sleep Disorders Diagnosis and Treatment Center of Hangzhou Seventh People’s Hospital. The center uses the “Good Sleep 365” smartphone platform (Hangzhou Xipu Diagnostic Co., Ltd., version 4.8.0), which has separate patient and clinician interfaces for pre-visit assessment and post-visit follow-up. Psychological assessment modules in the patient app include sleep quality, anxiety, depressive symptoms, daytime sleepiness, somatic symptoms, and cognitive function; trained staff in the clinic instruct patients to complete regular self-assessments. Clinicians enroll patients into the appropriate diagnostic category and monitor their assessments through the clinician interface. The study protocol was reviewed and approved by the Ethics Committee of Hangzhou Seventh People’s Hospital (approval No. 2024-044), which waived the requirement for individual informed consent because only anonymized data were analyzed. All procedures complied with the Declaration of Helsinki, and de-identified data were stored and managed by authorized personnel only.

From 1 November 2017 to 10 October 2024, we consecutively screened patients with MDD who visited the Sleep Disorders Diagnosis and Treatment Center and completed smartphone-based assessments via the “Good Sleep 365” app. All 321 eligible participants were included in the descriptive and correlation analyses, whereas predictive modeling was restricted to the 233 participants who completed PHQ-9 and PDQ-D-20 assessments at all scheduled follow-up visits. Inclusion criteria were: (1) meeting the International Classification of Diseases, 10th Revision (ICD-10) diagnostic criteria for a depressive episode; (2) age 18–65 years; (3) ability to use the “Good Sleep 365” app; and (4) at baseline, completion of the PDQ-D-20 with a PHQ-9 score ≥10, with PHQ-9 and PDQ-D-20 completed again at weeks 2, 4, 6, and 8. Exclusion criteria were: (1) comorbid organic brain disease, severe endocrine disease, primary cognitive disorders (including mild cognitive impairment, dementia, or other cognitive impairment syndromes), prodromal symptoms of neurodegenerative diseases (e.g., Parkinson’s disease, frontotemporal dementia), or other organic brain disorders that may independently cause cognitive impairment; (2) comorbid schizophrenia, intellectual disability, bipolar disorder, or other psychiatric disorders; (3) current alcohol or other psychoactive substance abuse; and (4) receipt of modified electroconvulsive therapy (MECT) within the past 6 months. In routine practice at this clinic, patients with MDD are treated according to national clinical guidelines, mainly with antidepressant pharmacotherapy, often combined with hypnotic agents or psychotherapy when indicated (14). However, information on the exact antidepressant regimen, dose adjustments and additional physical or psychological interventions was not systematically available in the retrospective database and therefore was not included in the present analyses.

### 2.2 Clinical and psychometric assessments

A self-developed questionnaire was used to collect demographic and clinical information, including sex, age, duration of illness, family history of psychiatric disorders, and level of education. Depressive symptoms were assessed with the PHQ-9. The PHQ-9 is brief, easy to administer, and has good internal consistency. It covers nine core symptoms of depression (low mood, loss of interest, sleep disturbances, appetite changes, fatigue, feelings of worthlessness or excessive guilt, psychomotor changes, concentration difficulties, and suicidal ideation). Each item is scored from 0 to 3, yielding a total score from 0 to 27, with higher scores indicating more severe depressive symptoms. Commonly used severity categories are: 0–4, minimal; 5–9, mild; 10–14, moderate; 15–19, moderately severe; and ≥20, severe depression. The Chinese version of the PHQ-9 has demonstrated good reliability and validity in community and clinical samples in China and is widely used as a screening tool for depression (15).

Subjective cognitive dysfunction was assessed with the PDQ-D-20, which has good reliability and validity. The PDQ-D-20 includes 20 items covering four cognitive domains: attention/concentration, retrospective memory, prospective memory, and planning/organization. Each item is scored from 0 to 4, with total scores ranging from 0 to 80; higher scores indicate more severe cognitive impairment. The Chinese version of the PDQ-D-20 has been psychometrically validated in patients with MDD in China and is suitable for evaluating perceived cognitive deficits in this population (9). In this study, PDQ-D-20 was administered at baseline and at weeks 2, 4, 6, and 8 using the same standardized instructions through the smartphone app; at each time point, participants were instructed to respond according to their experience in the previous two weeks, and the app did not display previous scores or feedback, which helped to reduce potential testing interference across repeated assessments.

### 2.3 Statistical analysis

Continuous variables were summarized as mean and standard deviation, and categorical variables as number and percentage. Baseline differences between response and non-response groups were examined with the t test for continuous variables and the chi-square test or Fisher’s exact test for categorical variables. Correlations between PHQ-9 total scores and PDQ-D-20 total and domain scores at each time point were analyzed with Pearson correlation. The reduction rate of PHQ-9 total score from baseline to weeks 4, 6 and 8 was calculated and its association with baseline PDQ-D-20 scores was also examined.

For the prediction of antidepressant response, response was defined as at least a 50 percent reduction in PHQ-9 total score from baseline to week 8 and smaller reductions were classified as non-response. Predictive modeling was carried out in the 233 participants who completed all scheduled PHQ-9 and PDQ-D-20 assessments. Three hierarchical logistic regression models were fitted. Model 1 included baseline PDQ-D-20 total score. Model 2 further included age and sex. Model 3 additionally incorporated baseline PHQ-9 total score, baseline GAD-7 score, baseline PSQI score, education level, occupation and illness duration. For each model we calculated odds ratios with 95 percent confidence intervals and examined variance inflation factors for all predictors to assess multicollinearity. Discrimination was evaluated by the area under the receiver operating characteristic curve, calibration by comparing predicted and observed probabilities, and decision curve analysis was used to assess clinical usefulness. A nomogram was built based on the fully adjusted model, and areas under the curves were compared between models with DeLong’s test.

## 3 Results

### 3.1 Baseline demographic and clinical characteristics

**Figure 1:**
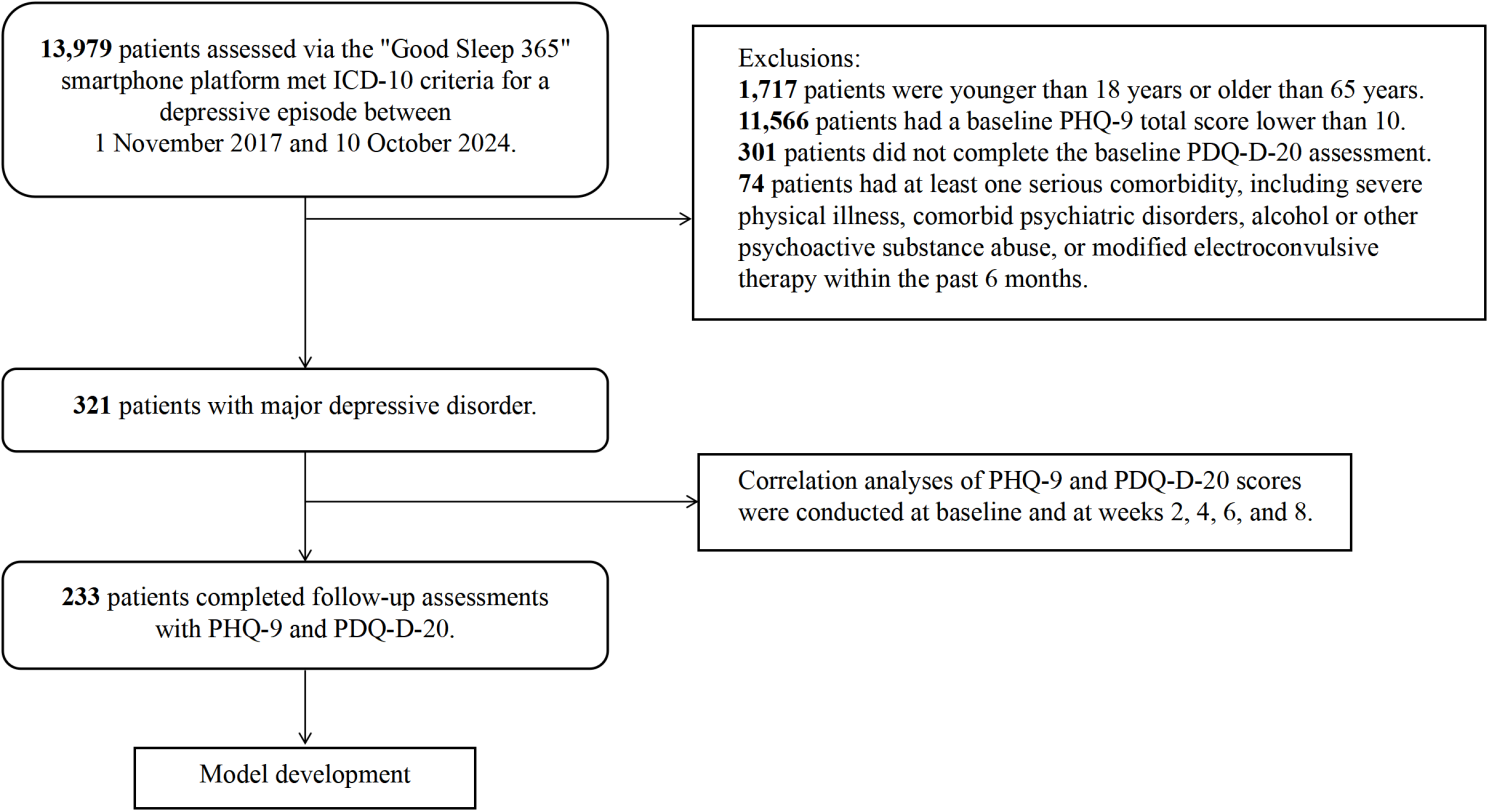
Study flowchart.

A total of 321 patients were included in the study. There were 84 men and 237 women, and the mean age was 36.32 ± 12.88 years. With regard to education, 22 participants had completed postgraduate education, 150 had completed college or university, 116 had completed junior high school or high school, and 33 had completed primary school. The flow of participant screening, inclusion and follow-up is shown in **Figure 1**. Among the 321 patients, 233 completed all scheduled PHQ-9 and PDQ-D-20 assessments over the 8-week period and were included in the predictive modeling analyses. Baseline demographic and clinical characteristics according to antidepressant treatment response status are presented in **Table 1**. In general, responders and non-responders did not show marked differences in age, sex, education level, occupation or illness duration.

**Table 1:** Baseline demographic and clinical characteristics of patients with major depressive disorder according to antidepressant treatment response.

Variable	Responders	Non-responders	P-value
n	178	55	
Age, mean ± SD	43.48±12.85	35.11±11.61	0.048
Gender-Female, n (%)	127(71.35)	41(74.54)	0.073
Occupation			0.651
Retired, n (%)	3(1.69)	1(1.82)	
Employee, n (%)	28(15.73)	9(16.36)	
Professional and Technical Personnel, n (%)	33(15.54)	8(14.55)	
Worker, n (%)	8(4.49)	3(5.45)	
Farmer, n (%)	3(1.69)	1(1.82)	
Freelancer, n (%)	67(37.64)	22(40.00)	
Unemployed, n (%)	23(12.92)	3(5.45)	
Student, n (%)	13(7.30)	8(14.55)	
Illness duration			0.131
<1 month, n (%)	23(12.92)	9(16.36)	
1 - 3 months, n (%)	48(26.97)	9(16.36)	
3 - 6 months, n (%)	24(13.48)	8(14.55)	
6 - 12 months, n (%)	31(17.42)	5(9.10)	
1-3 years, n (%)	16(8.99)	4(7.27)	
3-5 years, n (%)	12(6.74)	9(16.36)	
5-10 years, n (%)	24(13.48)	11(20.00)	
Education			0.602
Primary school, n (%)	16(8.99)	4(7.27)	
Junior high / high school, n (%)	80(44.94)	23(41.82)	
College / university, n (%)	69(38.77)	26(47.27)	
Postgraduate, n (%)	13(7.30)	2(3.64)	
Family history of psychiatric disorder, n (%)	41(26.97)	11(23.91)	0.824
Baseline PHQ-9, mean ± SD	19.03±4.39	17.89±4.44	0.614
Baseline GAD-7, mean ± SD	14.92±5.43	12.98±5.51	0.263
Baseline PSQI, mean ± SD	15.89±3.81	15.74±3.93	0.366
Baseline PDQ-D-20 total, mean ± SD	23.96±18.77	30.63±17.75	0.001
Baseline PDQ-D-20 attention/concentration, mean ± SD	6.88±6.08	8.96±5.55	0.001
Baseline PDQ-D-20 retrospective memory, mean ± SD	6.68±5.28	8.84±4.66	<0.001
Baseline PDQ-D-20 prospective memory, mean ± SD	4.47±4.39	6.87±5.10	<0.001
Baseline PDQ-D-20 planning/organization, mean ± SD	4.19±3.99	5.59±4.07	0.001

PHQ-9, Patient Health Questionnaire-9; GAD-7, Generalized Anxiety Disorder-7; PSQI, Pittsburgh Sleep Quality Index; PDQ-D-20, Patient-reported Cognitive Deficits Questionnaire-Depression 20.

### 3.2 Associations between depressive symptoms and subjective cognitive dysfunction

**Figure 2:**
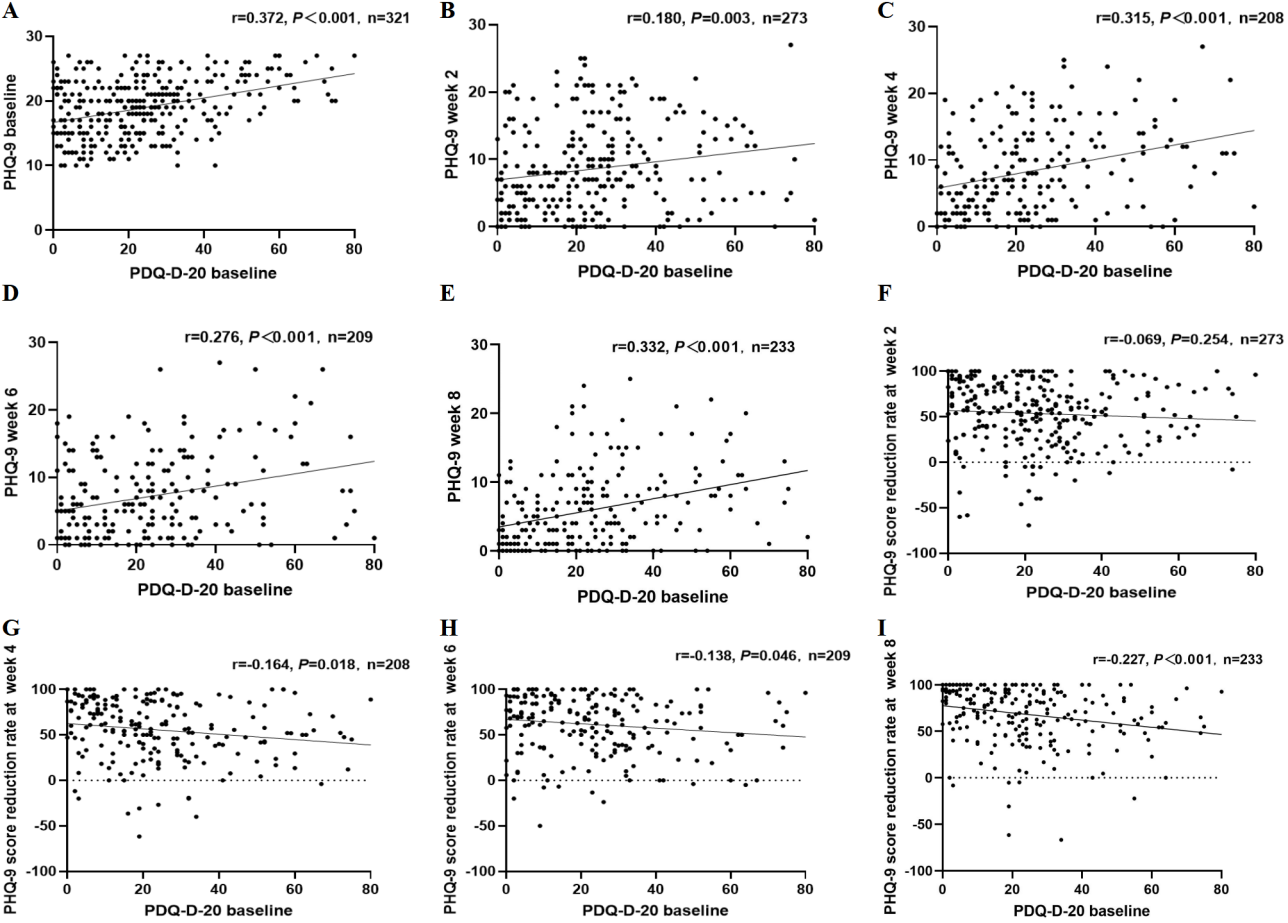
Relationships between depressive symptoms and subjective cognitive function at baseline and during 8-week follow-up. **(A)** Baseline correlation between PHQ-9 total score and PDQ-D-20 total score. **(B–E)** Correlations between PHQ-9 total score and PDQ-D-20 total score at weeks 2, 4, 6, and 8, respectively. **(F–I)** Correlations between baseline PDQ-D-20 domain scores (attention/concentration, retrospective memory, prospective memory, planning/organization) and the reduction rate of PHQ-9 total score over 8 weeks.

At baseline, PHQ-9 total scores showed a positive correlation with PDQ-D-20 total scores, indicating that patients with more severe depressive symptoms reported more pronounced subjective cognitive difficulties. Similar positive correlations between PHQ-9 and PDQ-D-20 total scores were observed at weeks 2, 4, 6 and 8 of follow-up. These relationships are illustrated in **Figures 2A–E**.

When PDQ-D-20 domain scores were considered, PHQ-9 total scores were positively correlated with impairments in attention and concentration, retrospective memory, prospective memory, and planning and organization at baseline and at subsequent assessments. Patients with higher depressive symptom scores tended to report worse functioning across all four cognitive domains. Detailed correlation coefficients for each time point and domain are shown in **Table 2**.

**Table 2:** Correlation between depressive symptoms and PDQ-D-20 factors.

Variable		PHQ9 baseline	PHQ9 2w	PHQ9 4w	PHQ9 6w	PHQ9 8w
PDQD20 baseline total score	r	0.372	0.18	0.315	0.276	0.334
	*p*	<0.001	0.003	<0.001	<0.001	<0.001
PDQD20 Baseline_Attention	r	0.334	0.213	0.293	0.227	0.275
	*p*	<0.001	<0.001	<0.001	0.001	<0.001
PDQD20 baseline_retrospective memory	r	0.362	0.185	0.296	0.246	0.32
	*p*	<0.001	0.002	<0.001	<0.001	<0.001
PDQD20 baseline_prospective memory	r	0.3	0.09	0.254	0.232	0.269
	*p*	<0.001	0.137	<0.001	0.001	<0.001
PDQD20 Baseline_Program Organizational Capacity	r	0.316	0.145	0.287	0.311	0.356
	*p*	<0.001	0.017	<0.001	<0.001	<0.001

### 3.3 Baseline subjective cognitive domains and improvement in depressive symptoms

Over the 8-week observation period, PHQ-9 total scores decreased on average, indicating an overall improvement in depressive symptoms. Baseline PDQ-D-20 total scores were inversely correlated with the reduction rate of PHQ-9 total scores at weeks 4, 6 and 8. Patients who reported better overall cognitive function at baseline generally experienced greater improvement in depressive symptoms.

At the level of cognitive domains, higher baseline scores for attention and concentration, retrospective memory, prospective memory, and planning and organization were all associated with smaller reductions in PHQ-9 total scores. Among these domains, poorer baseline planning and organizational ability was particularly associated with less improvement in depressive symptoms. These associations are summarized in **Figures 2F–I**; **Table 3**.

**Table 3:** Correlations between baseline PDQ-D-20 scores and the 8-week reduction rate of PHQ-9 total score.

Variable		PHQ9 2w penalty rate	PHQ9 4w penalty rate	PHQ9 6w penalty rate	PHQ9 8w penalty rate
PDQD20 Baseline total score	r	-0.069	-0.164	-0.138	-0.229
	*p*	0.254	0.018	0.046	<0.001
PDQD20 Baseline Attention	r	-0.114	-0.167	-0.116	-0.191
	*p*	0.06	0.016	0.093	0.003
PDQD20 Baseline retrospective memory	r	-0.069	-0.151	-0.115	-0.217
	*p*	0.253	0.03	0.097	0.001
PDQD20 Baseline prospective memory	r	-0.005	-0.11	-0.108	-0.182
	*p*	0.94	0.113	0.121	0.005
PDQD20 Baseline program organizational capacity	r	-0.054	-0.157	-0.172	-0.252
	*p*	0.377	0.024	0.013	<0.001

### 3.4 Performance of logistic regression models for predicting antidepressant treatment response

In the subgroup of 233 participants who completed all follow-up assessments, three hierarchical logistic regression models were fitted to predict antidepressant treatment response at week 8, defined as at least a 50 percent reduction in PHQ-9 total score from baseline. Model 1 included baseline PDQ-D-20 total score. Model 2 further included age and sex. Model 3 additionally incorporated baseline PHQ-9, baseline GAD-7, baseline PSQI, education level, occupation and illness duration.

Across the three models, higher baseline PDQ-D-20 total scores were associated with a lower probability of treatment response. As more variables were added, model discrimination improved. Model 1 showed modest performance, whereas Model 2 achieved a higher area under the receiver operating characteristic curve. The fully adjusted Model 3 provided the best overall performance, with an area under the curve of about 0.91 and an accuracy of about 0.82, together with a favourable balance between precision and recall. Pairwise DeLong tests indicated that both Model 2 and Model 3 outperformed Model 1, and that Model 3 performed better than Model 2.

For Model 3, the calibration curve showed reasonable agreement between predicted and observed probabilities of treatment response, and decision curve analysis suggested a clear net clinical benefit across a wide range of threshold probabilities. A nomogram based on Model 3 was constructed to allow individual estimation of the probability of achieving treatment response using baseline cognitive, symptom and demographic variables. The main performance metrics of the three models are reported in **Table 4**, variance inflation factors for all predictors are shown in **Table 1**, and pairwise comparisons of areas under the curves are summarized in **Table 2**.

**Table 4:** Performance metrics of the three logistic regression models for predicting antidepressant treatment response.

Metric	Model 1	Model 2	Model 3
AUC	0.664	0.831	0.909
Accuracy	0.537	0.761	0.820
Precision	1.000	0.812	0.839
Recall	0.073	0.680	0.792
F1-score	0.136	0.740	0.815

## 4 Discussion

In this outpatient sample of adults with MDD recruited from a sleep-disorders clinic and followed via a smartphone platform, we found that more severe subjective cognitive dysfunction was consistently associated with higher depressive symptom severity at baseline and across eight weeks of routine treatment. Baseline PDQ-D-20 total and domain scores were also related to subsequent improvement in PHQ-9 scores, and higher scores in domains such as planning and organization were linked to a lower probability of achieving a 50% reduction in depressive symptoms. In addition, our logistic regression models showed that baseline subjective cognitive complaints can contribute to the prediction of antidepressant treatment response, especially when combined with demographic and other clinical variables. These findings suggest that subjective cognitive symptoms are not only concurrent correlates of depressive severity but may also be clinically relevant markers of short-term treatment outcome.

The pattern of cross-sectional and longitudinal associations observed in this study is broadly in line with previous work showing that cognitive symptoms are common in MDD and closely related to depressive symptom burden, everyday functioning and quality of life (10–13). Meta-analyses of objective neuropsychological performance have shown that patients with MDD exhibit impairments in attention, processing speed, memory and executive functions, and that these deficits often persist beyond symptomatic remission (10, 12). Large observational studies using self-report instruments similar to the PDQ-D-20 have further reported that subjective cognitive complaints are associated with poorer functional outcomes and a higher risk of relapse (11, 13). Our results extend this literature by demonstrating similar associations in a Chinese outpatient cohort with prominent sleep complaints, assessed repeatedly over eight weeks via a smartphone-based platform rather than in traditional clinic-based research settings.

**Figure 3:**
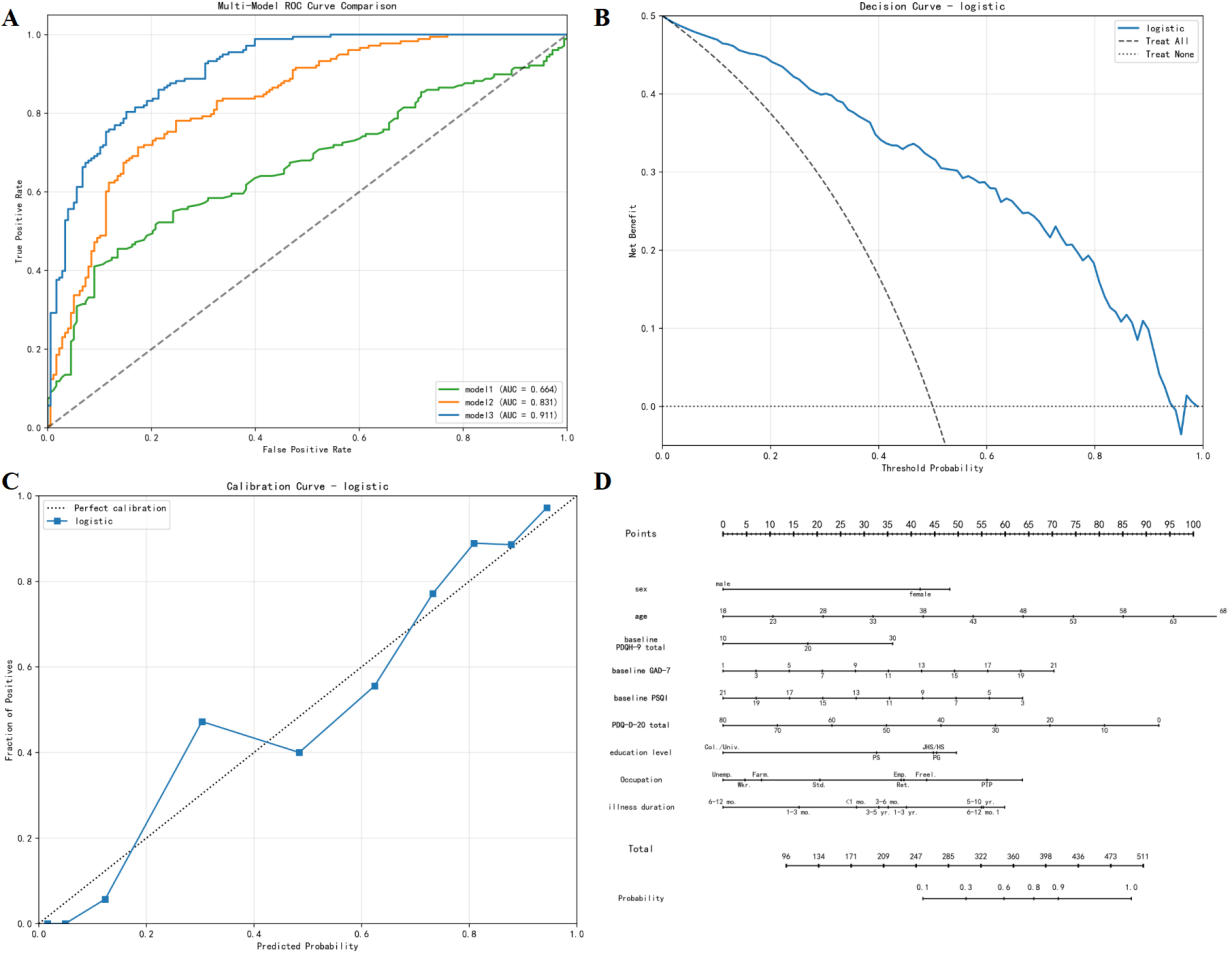
Prediction model for antidepressant treatment response in patients with major depressive disorder. **(A)** ROC curve of the logistic model for discriminating responders (≥50% reduction in PHQ-9 total score from baseline) from non-responders (<50% reduction). **(B)** Decision curve analysis showing the net clinical benefit of using the model across a range of threshold probabilities compared with the treat-all and treat-none strategies. **(C)** Calibration curve illustrating the agreement between predicted and observed probabilities of treatment response. **(D)** Nomogram based on the logistic regression model.

The predictive modeling results add an additional layer of clinical relevance. When PDQ-D-20 total score was used alone, the model showed only modest discrimination between responders and non-responders. As demographic variables and other baseline symptom measures were added, model performance improved, and the full multivariable model showed good discrimination and acceptable calibration, with decision-curve analysis indicating clear net clinical benefit across a range of threshold probabilities (**Table 4**; **Figure 3**). Pairwise DeLong tests confirmed that the full model performed significantly better than the PDQ-D-20–only model and the model including PDQ-D-20 plus a limited set of covariates (**Table 2**). These findings suggest that subjective cognitive complaints carry prognostic information, but their predictive value is enhanced when jointly considered with baseline depressive severity, anxiety symptoms, sleep quality, educational attainment, illness duration and occupational status.

Our findings can be interpreted within current neurobiological models of depression and cognition. Chronic stress and depressive episodes are thought to impair synaptic plasticity and neurogenesis, particularly in prefrontal and hippocampal circuits that subserve executive control, memory and emotion regulation (16). Reductions in brain-derived neurotrophic factor (BDNF) signaling and related neurotrophic pathways may contribute to both mood dysregulation and enduring cognitive inefficiency, even after partial symptomatic improvement (16). In parallel, converging evidence points to cortical GABAergic interneuron dysfunction and disturbed excitatory–inhibitory balance in key cognitive and affective networks in depression (17). Such circuit-level alterations may manifest clinically as difficulties in sustaining attention, holding information in mind and planning goal-directed behavior, which are captured by PDQ-D-20 domains. Patients who endorse more severe subjective deficits at baseline may therefore have more entrenched network dysfunction and reduced capacity for neuroplastic recovery under standard antidepressant treatment, which could explain the weaker symptom improvement observed in this subgroup.

The clinical implications of these results complement emerging evidence on cognitive remediation and cognitive rehabilitation in MDD. Recent systematic reviews and meta-analyses have shown that cognitive remediation produces moderate improvements in global cognition and executive functioning in adults with depression, with small but significant benefits for depressive symptoms and everyday functioning (18, 19). A more recent multicenter randomized controlled trial conducted in routine clinical settings reported that computerized cognitive remediation improved both objective and subjective cognitive performance in patients with MDD, alongside favourable effects on clinical outcomes (20). Against this background, our finding that baseline subjective cognitive dysfunction is associated with poorer short-term antidepressant response supports the idea that systematic assessment of cognitive complaints could help identify patients who might benefit from adjunctive cognitive remediation or other pro-cognitive interventions, rather than relying on pharmacotherapy alone.

The present study also has methodological strengths. First, it used repeated assessments of depressive symptoms and subjective cognition over eight weeks, allowing us to examine both cross-sectional and longitudinal relationships rather than relying on a single time point. Second, data were collected through a widely used smartphone platform in a specialized sleep-disorders clinic, which increases ecological validity and reflects routine outpatient clinical practice in China. Third, by combining correlation analyses with multivariable logistic regression and complementary evaluation tools such as ROC curves, calibration plots, decision-curve analysis and a nomogram, we were able to move beyond simple association toward a more clinically interpretable prediction framework.

Several limitations should be acknowledged. First, all cognitive and symptom measures in this study were based on self-report scales rather than objective neuropsychological tests, which may introduce reporting bias and limit our ability to disentangle subjective from objective cognitive deficits. Repeated administration of the same self-report scales over eight weeks may also have introduced expectancy or learning effects, although we attempted to minimize this by providing no feedback on previous scores. Second, although we included basic demographic and clinical variables, we lacked detailed information on antidepressant class, dose, switching and augmentation strategies, as well as on psychotherapy and other non-pharmacological treatments, which may have influenced both cognitive trajectories and treatment response. Third, the analytic cohort was drawn from a single center specializing in sleep-disorders and consisted of sleep-disturbed patients who were able and willing to use a smartphone application, which may limit generalizability to MDD patients without comorbid sleep issues, to other clinical settings or to individuals with more severe illness or lower digital literacy. Fourth, the predictive models were developed and internally evaluated in the same dataset, so the risk of overfitting cannot be fully excluded despite acceptable variance inflation factors and model diagnostics; external validation in independent samples is required before the nomogram can be adopted in routine care. Finally, the follow-up period was limited to eight weeks, and we were unable to assess longer-term cognitive outcomes, functional recovery or recurrence of depressive episodes.

Taken together, this study provides clinical evidence from a Chinese outpatient setting that subjective cognitive dysfunction is closely linked to depressive symptom severity and short-term treatment response in Chinese outpatients with MDD, and that incorporating baseline cognitive complaints into multivariable models improves the prediction of antidepressant response. These findings support routine assessment of subjective cognition in clinical practice and suggest that future work should integrate objective cognitive testing, neurobiological markers and cognitive remediation strategies to better understand and treat the cognitive dimension of depression.

## 5 Conclusion

In this smartphone-based cohort of Chinese outpatients with major depressive disorder attending a sleep-disorders clinic, subjective cognitive dysfunction measured by PDQ-D-20 was closely related to depressive symptom severity, and higher baseline scores predicted a lower probability of achieving a PHQ-9 treatment response at eight weeks. A multivariable logistic regression model that combined PDQ-D-20, symptom scales and basic demographic and clinical variables showed good discrimination and clinical usefulness for predicting treatment response. Routine assessment of subjective cognition may help identify patients at higher risk of suboptimal antidepressant outcomes and support more individualized monitoring and intervention, a finding that warrants confirmation in prospective studies with longer follow-up.

## Data Availability

The raw data supporting the conclusions of this article will be made available by the authors, without undue reservation.
